# Effect of Sodium Selenite, Selenium Yeast, and Bacterial Enriched Protein on Chicken Egg Yolk Color, Antioxidant Profiles, and Oxidative Stability

**DOI:** 10.3390/foods10040871

**Published:** 2021-04-16

**Authors:** Aliyu Ibrahim Muhammad, Dalia Abd Alla Mohamed, Loh Teck Chwen, Henny Akit, Anjas Asmara Samsudin

**Affiliations:** 1 Department of Animal Science, Faculty of Agriculture, Universiti Putra Malaysia, Serdang 43400, Selangor, Malaysia; aliyudambam@gmail.com (A.I.M.); tcloh@upm.edu.my (L.T.C.); henny@upm.edu.my (H.A.); 2 Department of Animal Science, Faculty of Agriculture, Federal University Dutse, Dutse P.M.B. 7156, Jigawa State, Nigeria; 3 Department of Animal Nutrition, Faculty of Animal Production, University of Khartoum, P.O. Box 321, Khartoum 11115, Sudan; dalida983@yahoo.com

**Keywords:** antioxidant profile, bacterial selenoproteins, chicken egg, egg yolk color, oxidative stability, selenium, *Stenotrophomonas maltophilia-*ADS18

## Abstract

The chicken egg is one of nature’s flawlessly preserved biological products, recognized as an excellent source of nutrients for humans. Selenium (Se) is an essential micro-element that plays a key role in biological processes. Organic selenium can be produced biologically by the microbial reduction of inorganic Se (sodium selenite). Therefore, the possibility of integrating Se enriched bacteria as a supplement in poultry feed can provide an interesting source of organic Se, thereby offering health-related advantages to humans. In this study, bacterial selenoproteins from *Stenotrophomonas maltophilia* was used as a dietary supplement with other Se sources in Lohman brown Classic laying hens to study the egg yolk color, egg yolk and breast antioxidant profile, oxidative stability, and storage effect for fresh and stored egg yolk at 4 ± 2 °C for 14-days. The results showed that dietary Se supplementation significantly (*p* < 0.05) improved egg yolk color, the antioxidant profile of egg yolk, and breast meat (total carotenoid and phenol content). When the Se treated groups were compared to control groups, there was a significant (*p* < 0.05) decrease in total cholesterol in fresh and stored egg yolk and breast muscle. In hens that were fed ADS18-Se, the primary oxidation products (MDA) concentrations in the eggs, breast, and thigh muscle, and plasma were significantly (*p* < 0.05) lower. However, the MDA content increased (*p* < 0.05) with an extended storage time in egg yolk. In comparison to inorganic Se and basal diets, egg yolk from hens fed organic Se remained fresh for two weeks. The egg yolk color, antioxidant profile, and oxidative status of egg yolk and tissue improve with dietary Se organic supplementation (ADS18 > Se-Yeast). The source of supplemented organic Se is critical for egg enrichment and antioxidant properties. As a result, ‘‘functional eggs’’ enriched with organic Se becomes possible to produce.

## 1. Introduction

Shell eggs [1] are among the few foods that are consumed by people of all ages, regardless of religion or ethnicity worldwide [2]. The chicken egg is one of nature’s flawlessly preserved biological materials [1], as well as a delectable and easy-to-digest source of excellent nutrients for humans [3,4]. Egg yolk contains nutritive and non-nutritive compounds that are beneficial to human health, according to a study by Attia et al. [5]. However, Elkin [6] recommends against consuming shell eggs because they have been shown to elevate cholesterol levels and are related to hypercholesterolemia and risk factors for coronary heart disease, so they should be consumed in moderation [5]. Weggemans et al. [7] reported an increase in blood cholesterol concentration, which was believed to be the result of high egg intake and which may contribute to various cardiovascular risks. However, since the scientific evidence linking dietary cholesterol (from eggs) to the risk of cardiovascular diseases is more or less insubstantial, Qureshi et al. [8] found no correlation between egg intake and increase in blood cholesterol concentrations, implying that the cardiovascular risk is largely dependent on increase in serum total and LDL-cholesterol [8]. It was also reported that since eggs increase satiety, they are now being used as a weight-loss treatment. Qureshi et al. [8] found that eating one egg per day does not increase the risk of stroke or ischemic stroke, and that the belief that higher egg intake among diabetics is linked to an increased risk of coronary artery disease warrants further research. The chicken egg is considered as a functional food with an edible portion made up of water (74%), high-quality proteins (12%), lipids (12% of polyunsaturated fatty acids (PUFA) and phospholipids), carbohydrate (<1%), as well as vitamins and minerals [9,10]. Antioxidant and nutraceuticals properties were found in egg proteins (such as ovalbumin, ovotransferrin, phosvitin), egg lipids (phospholipids), micronutrients such as vitamin A and E, selenium, and carotenoids [11,12]. Furthermore, the chicken egg can be also enriched with antioxidants (natural or synthetic) via poultry feed manipulations [13].

Selenium is an important micro-mineral that acts primarily as an antioxidant through selenoproteins such as glutathione peroxidase (GPX), thioredoxin reductases (TrxR), and selenoprotein P (Sepp1) [14]. By catalyzing the reduction of hydrogen peroxide and hydroperoxide activities, they protect cells and tissues from oxidative damage [15,16]. Surai et al. [17] highlighted that selenium-enriched eggs provided up to 50% of the daily Se requirements and were high in nutrients like omega 3, vitamin E, and D, selenium, and antioxidants like lutein. Pham-huy et al. [18] highlighted the potential role of antioxidants in disease prevention and health maintenance. Egg storage time can cause chemical changes in the eggs, deteriorating their quality [19]. Egg yolk is an excellent source of nutrients for humans [20], and abundant in polyunsaturated fatty acids that are prone to oxidation, with a coefficient of digestion comparable to milk and a superior biological value of proteins [13]. Progressive storage time changes the physicochemical properties of eggs [21]. The concentration and ratio of saturated and unsaturated fatty acids changes due to oxidation, particularly during storage.

Lipid peroxidation is a process that deteriorates egg quality, specifically yolk lipid stability during storage. It degrades the nutritional quality of an egg causing undesirable changes in taste, flavor, odor, color depreciation, and toxic substances [21]. The prevention of these effects can be achieved by supplementing hen feed with selenium as an antioxidants source. Antioxidants including selenium and vitamin E play a crucial role in anti-lipid oxidation protective processes [22]. Human Se intake is often lower than the recommended daily allowance [23], necessitating its consumption via other food sources. Poultry eggs can be enriched or fortified by adding selenium (inorganic or organic) compounds to the hen’s diets [24]. Organic Se, such as Se-enriched yeast, Se-proteinate produced enzymatically by hydrolyzed soy protein, nano-Se, and Se-amino acids [25], has recently become available. *Saccharomyces cerevisiae* has been established as the main strain used for aerobic fermentation in Se-enriched media with sodium selenite as Se source, which accumulates and incorporates into organic Se-containing compounds to produce Se-yeast [26]. Organic Se supplements have several advantages over inorganic Se supplements, including increased egg Se concentration, improved oxidative stability, and a better fatty acid profile of the stored egg content [13,25].

There is also a new organic Se-enriched dietary source derived from bacteria (*Stenotrophomonas maltophilia* (ADS18)) [27], which may provide health-related benefits to humans through animal products. To our knowledge, no research has been done on its (Se-enriched bacterial proteins of ADS18) use in layers. Therefore, this study aims to examine the effects of different dietary Se sources (sodium selenite, selenium yeast, *Stenotrophomonas maltophilia* enriched bacterial protein (ADS18) on egg yolk color, egg yolk, and breast meat antioxidant profile, total antioxidant capacity (TAC), and oxidative stability for fresh and stored egg yolk at 4 ± 2 °C for 14 days.

## 2. Materials and Methods

### 2.1. Ethical Statements

All procedures involving animal care, handling, and sampling were performed in compliance with the guidelines and regulations and were approved by the Universiti Putra Malaysia’s Institutional Animal Care and Use Committee (UPM/IACUC/AUP-R063/2018) before the commencement of the research.

### 2.2. Experimental Hens, Design, and Diets

A commercial farm provided one hundred and forty-four 18-week-old Lohman Brown-Classic hens (initial live weight 1714 ± 185 g). At the Poultry Unit, Universiti Putra Malaysia, Serdang, the hens were raised in an open ventilated layer-house and A-shape two-tier stainless-steel cages with one bird per cage. The cage measured 30 cm by 50 cm by 40 cm (width depth height) in size. Using FeedLIVE software, a corn and soya bean-meal basal layer diet was prepared according to the Nutrient Requirement of Poultry (NRC) for layers guideline [28] (Table 1). The hens were randomly divided into four groups (36 hens in each) at 23 weeks of age, and different forms of Se were added to standard diets. The treatments diets were designated as a group I (diets without Se supplementation), group II (with 0.3 mg/kg of inorganic Se in the form of sodium selenite, group III (with 0.3 mg/kg of organic Se from SelPlex (Altech Inc.) (SY), and group IV (with 0.3 mg/kg of organic Se from *Stenotrophomonas maltophilia* (bacterial organic Se)). The concentrations of selenium in the diets without Se supplementation, inorganic, organic Se-yeast, and bacterial organic Se were found to be 0.031, 0.312. 0.320, and 0. 339 mg/kg, respectively, using Inductively Coupled Plasma Mass Spectrometry (ICP.MS) as described previously [29,30,31]. Briefly, the samples were digested with analytical grade nitric acid (HNO_3_), 65%, (Sigma-Aldrich, St Louis, MO 63101, USA) and hydrogen peroxide (H_2_O_2_) (Emsure^®^ Iso, Merck, Darmstadt, Germany) on DigiPrep (SCP Science, Courtaboeuf, France). After cooling at room temperature, the digesta was diluted with water containing Rh and Ga as internal standard to a final volume of 19 mL. A Certified Reference Materials CRM 626 (European Commission) and a blank were analyzed along with each series of samples. The samples and standards were diluted with deionized water obtained by the Milli-Q System (18.2 MΩ cm; EMD Millipore, Darmstadt, Germany). Stock standard solutions of Ga with a concentration of 1000 mg L^-1^ were used to prepare the internal standard (Ga/In solution). The internal standard solution was diluted to 100 µg L^−1^. The quantification (external calibration) was carried out by preparing standard of 1, 10, 25, 50, 75, and 100 µg L^−1^ and adding the internal standard, with Ga/In in a final concentration of 2 µg L^−1^. A sample volume of 20 μL was used in each analysis. The ICP MS collision cell was pressurized with hydrogen and samples were introduced into the exit of the column which was directly coupled to the ICP-MS fitted with a MicroMist nebulizer and a cyclonic Scott double-pass spray chamber [31]. ^76^Se, ^77^Se, ^78^Se, and ^103^Rh were monitored, and ^82^Se was used for quantitative determination. The detection limit was 0.8 ng/mL, and the quantification limit was 4.0 nM, with a relative standard deviation (precision of repeated injections) of 2.2%. The procedure for bacterial selenoproteins from *S. maltophilia* production is described in the previous studies [27]. The hens were fed 120 g of feed a day per hen, and unlimited access to water. The ambient temperature during the experimental duration was about 30 ± 5 °C. The lightening schedule was practiced with a 16-h light and 8-h dark, with the light starting at 17:00 local time and according to the Lohman management guide [32]. The experiment took sixteen weeks to complete.

### 2.3. Blood, Breast, and Egg Yolk Sample Collection and Chemical Analysis

To collect blood and tissue samples, a total of twenty-four hens were selected randomly from each treatment (hen per each replicate) and slaughtered according to the Halal procedure, as described in the Malaysian Standard [33]. Blood samples (10 mL) were collected in BD Vacutainer^®^ Plus Plastic Serum Tubes (Becton Dickinson, Franklin Lakes, NJ, USA) via hen’s jugular vein. Blood samples were centrifuged at 3000 rpm for 10 min, after which the supernatant (serum) was harvested and stored at −80 °C for further analysis [34]. A part of the breast meat sample was snapped frozen in liquid nitrogen before being stored at −80 °C further assays.

Six representative eggs per replicate were randomly sampled on days 2, 12, and 14-d post storage of the experimental periods egg quality assay. The egg yolks of four hens per replication of the dietary treatment group were pooled; thus, instead of 36 yolk samples per treatment, 24 yolk samples per treatment were collected for each sampling. After that, the egg yolk samples were used to evaluate the antioxidant profile (total carotene, cholesterol, phenolic, and flavonoid content), oxidative stability-influencing status (antioxidant capacity, thiobarbituric acid reactive substances (TBARs)), and egg yolk color assay. Eggs laid and collected on the last day of the experiment were weighed and stored on an egg paper tray at 4 ± 2 °C for 14 days to determine shelf-life. Following the storage time, the eggs were subjected to the same analysis.

### 2.4. Egg Yolk Color Measurement

The color of samples (freeze-dried egg yolk) was measured in line with the Yolk Color Fan^®^ Scale DSM Yolk Color Fan (DSM Nutritional Products Europe, Wurmisweg 576, CH-4303 Kaiseraugst, Switzerland) starting with 1 as yellow and 15 for orange), and MINOLTA CR300 (Minolta Camera Co. Ltd., Osaka, Japan) was used as the color measuring device. This was per CIE (*Commission Internationale d’Eclairage*) Lab color System, L* (lightness: negative towards black and positive towards white). a* (redness: negative towards green and positive towards red) and b* (yellowness: negative towards blue and positive towards yellow). The device was calibrated by the reference tiles ‘’rose tile ‘’ (L* 44.88, a*25.99, b*6.67) and light source D-65 [35,36], with samples placed on the glass cup and measured three times. The average of the three corresponding readings was considered and analyzed statistically as the final values.

Egg chroma was calculated according to Omri et al. [34]:

C* = (a*2 + b*2)^1/2^ (1)

Hue was determined according to Bianchi et al. [37]:

H* = tan^−1^ b*/a* (2)

### 2.5. Antioxidant Profile Determination

#### 2.5.1. Determination of Total Carotene in Egg Yolk, Breast Tissue, and Feed Samples

The total carotenoid content of fresh, stored egg yolk, frozen breast tissue, and feed samples was determined using the method of Okonkwo [38] with slight modifications. Briefly, 1 g of homogenized (Wiggen Hauser^®^ D-500, Berlin, Germany) egg yolk was weighed in a conical flask. Cold acetone was added in two stages: 2 mL to make a smooth paste, followed by 8 mL for at least a minute. The solution was vortexed until it formed a homogenous mixture, and two-5 mL aliquots of acetone were used to rinse and re-extract it, accompanied by 1 mL deionized water. A 5 mL n-hexane was pipetted into new 15 mL falcon tubes, which the solution was transferred to and centrifuged at 3000 g for 10 min. The recovered acetone was filtered (equivalent to Whatman No. 4) and diluted to 100 mL. A spectrophotometer (Secomam, Domont, France) was used to measure the pigmentation of the egg yolk (n-hexane layer) at a wavelength (E1% 2500) of A_450 nm_.

(3) $$\mathrm{Conc}.\;\left(\mathrm{mg}/g\right)=\;\mathrm{Conc}.\;\left(\mathrm{mg}/g\right)=\frac{A\;\times \;\mathrm{Volume}\;\left(\mathrm{mL}\right)\times 104}{A10\%\;1\;\mathrm{cm}\;\times \;\mathrm{sample}\;\mathrm{weight}}$$

where A = absorbance, A^10^% 1 cm = 2592 to beta carotene (as a constant), Volume = 25 mL, Sample weight = weight of egg yolk, breast tissue, and feed samples

#### 2.5.2. Total Cholesterol (Spectroscopy AOAC Method)

The determination of cholesterol was carried out using the method described by [39]. First, 1 g of the sample was weighed into a 15 mL falcon tube with an addition of 3 mL 95% ethanol and 2 mL 50% potassium hydroxide, then homogenized (Wiggen Hauser^®^ D-500, Berlin, Germany) immediately for a few seconds. The homogenate was incubated in a water bath at 60 °C for 10 min before being removed and allowed to cool at room temperature. The homogenates were vortex/mixed vigorously with 5 mL of hexane. About 3 mL of deionized water was added, vortexed, and allowed to settle at room temperature for 15 min to enable complete phase separation. Around 2.5 mL of the upper phase (hexane layer) was taken and transferred into a clean glass tube, after which the hexane was evaporated to dryness under nitrogen gas flow at 60 °C. The residue was re-suspended with 4 mL o-phthalaldehyde reagent and left for 10 min at room temperature. Then, 2 mL of concentrated sulphuric acid was applied slowly and cautiously, vortex immediately. It was allowed for at least 10 min at room temperature before taking the absorbance at 550 nm against a blank (prepared without sample). The cholesterol standards (Sigma-Aldrich, L-4646, Merck KGaA, Darmstadt, Germany) had a concentrations of 0, 10, 20, 30, 40, 50, 60, 70, 80, 90, and 100 µg cholesterol per ml, according to the method described by [39].

#### 2.5.3. Total Phenol Determination in Egg Yolk and Breast Tissue

The total phenolic content of lyophilized fresh and stored egg yolk and meat tissue was determined using the Folin-Ciocalteu method [36,40], with all extraction and dilutions operations conducted in the dark. To determine the total phenols in egg yolk, approximately, 1.0 g yolk sample (lyophilized) was weighed and mixed with 1 M 80:20, *v*/*v* methanol/ HCl (10 mL), and the mixture was adjusted to pH = 1.5. The mixture was vortexed for 2 min and then centrifuged at 6000× *g* for 10 min at 4 °C. The supernatant was evaporated under vacuum rotary-evaporator at 35 °C. The residue was reconstituted by using 1 mL of methanol and filtered (0.22 µm pore size filter paper). The extract thus obtained was used for total phenols analaysis by Folin-Ciocalteu method [36,40]. Briefly, 1.0 mL (methanol extract) was homogenized with 0.5 mL of 2 *N* Folin-Ciocalteu reagent, then 2.5 mL (20% *w*/*v*) sodium carbonate (Na_2_CO_3_). After vortexing for a few seconds, the solution was set in a water bath and incubated at 60 °C for 40 min. A spectrophotometer (Secomam, Domont, France) was used to measure absorbance at 750 nm against a blank (distilled water), which was immediately cooled to room temperature. A standard curve of different concentrations using Gallic acid (0 to 140 µg /mL) was prepared to obtain a regression line in which sample absorbance was equated. The total phenolic content was calculated and expressed per gram of sample as mg equivalents gallic acid (EGA) (standard). The gallic acid standards (Sigma G7384) were prepared using the method of [41], using a solution with concentrations ranging from 20 to 140 ppm and in compliance with Beer’s Law at 750 nm.

#### 2.5.4. Determination of Flavonoid Content in Egg Yolk and Breast Tissue

The total flavonoid content was determined by the aluminum chloride method as reported [42]. Briefly, 0.1 g of fresh, stored egg yolk or diet samples were vortexed for 30 s before being collected in 5 mL diethyl in the dark. The homogenate was centrifuged for 15 min at 2000 rpm at 20 °C, with the supernatant collected for re-extraction. The pooled supernatant was evaporated at 40 °C in a water bath for 60 min (or drying by N gas) with tubes left uncovered. The residue was reconstituted by adding 5 mL of 80% methanol, vortex vigorously, and allowed for 5 h after which it was filtered (Whatman filter paper No. 4) and adjusted the filtrate with 50 mL deionized water. An aliquot of 2.5 mL was combined with 5% of 0.15 mL sodium nitrite (NaNO_2_), vortexed, and allow for 5 min. Thereafter, 0.15 mL of 10% aluminum chloride (AlCl_3_) was added. Six minutes later, 1 mL of NaOH (1N) and 1.2 mL of deionized water were added, and vortexed vigorously. The absorbance of the solution was measured at 510 nm against distilled water (blank). The flavonoid content was expressed as mg equivalents rutin (standard) per g of sample.

### 2.6. Oxidative Status Determination

#### 2.6.1. Total Antioxidant Capacity (TAC) by the Phosphomolybdenum Method

The total antioxidant capacity assay is a spectroscopic method for the quantitative determination of antioxidant activity by the formation of phosphomolybdenum complex according to the procedure described by Benzie et al. [43] and Omri et al. [36]. The fresh, stored, and breast tissue samples weighing around 0.5 g were homogenized and vortex vigorously in 10 mL of 2% sodium chloride (NaCl). An aliquot of 0.1 mL of sample solution was pipetted and adjust the volume to 2 mL (1.9 mL) with distilled water. After that, 2 mL of phosphomolybdenum reagent (28 mM sodium phosphate and 4 mM ammonium molybdate in 0.6 M sulphuric acid) was added. The reaction mixture is heated at 95 °C for 90 min, with the tubes capped and encapsulated in aluminum foil to prevent direct light exposure. After the thermal treatment, the reaction mixture was cooled at room temperature for some time and the absorbance was measured at 695 nm (A_695 nm_) using a UV- visible spectrophotometer (Secomam, Domont, France) against 2% NaCl (blank). The antioxidant capacity is expressed as mg equivalents of ascorbic acid (EAA) (standard) per g of sample.

#### 2.6.2. Antioxidant Activity (GAE/g) by Ferric Reducing Antioxidant Power (FRAP) Assay

The ferric reducing property of the extracts was determined using the assay described [36,43,44] with slight modifications. For this assay, aqueous solutions of the samples (egg yolk) prepared as mentioned above were used. Egg yolk extract (0.15 mL) was mixed with 2.4 mL of distilled water, 0.45 mL ethanol, 0.75 mL hydrochloric acid (HCl), 0.75 mL of 1% potassium ferricyanide (C_6_N_6_FeK_3_), 0.25 mL of 1% sodium dodecyl sulfate (NaC1_2_H_25_SO_4_) and 0.25 mL of 0.2% ferric chloride (FeCl_3_). The tubes containing the mixture were capped and incubated at 50 °C for 20 min, then allowed to cool at room temperature before measuring absorbance at 750 nm. The antioxidant capacity is expressed as mg Equivalents Gallic Acid (EGA) (standard) per g of sample.

#### 2.6.3. Thiobarbituric Acid Reactive Substances (TBARs), µg MDA/g

The malondialdehyde (MDA) assay method described by Botsoglou et al. [45] was used for lipid peroxidation of fresh, stored egg yolk, breast, thigh tissue, and serum. Measurements of the breast muscle were taken on the 0, 1, and 5 days of refrigerated samples at 4 °C. TBARs values were used to quantify lipid oxidation according to the modified method of Omri et al. [36]. Briefly, 1.0 g (meat and egg yolk) of the sample was weighed, and 4 mL 0.15 M potassium chloride was added and homogenized (Ultraturrax at 6000 rpm) for a minute. The sample solution was kept on ice to prevent further reaction. Each sample received a TBARS (fresh prepared) solution containing 0.8% thiobarbituric acid, 8.1% sodium dodecyl sulfate, and 7.0 mM butylated hydroxytoluene, which was added, mixed, and heated in a water bath at 95 °C for 60 min until pink color appeared. Thereafter, the samples were cooled under running water, 3 mL of n-butanol was added to the extracts and vortexed for 30 to 60 s. The mixture was centrifuged at 5000 rpm for 10 min at room temperature. The upper layer (n-butanol layer) was separated, and the absorbance at 532 nm was measured against pure butanol (as blank) using a UV-visible spectrophotometer (Secomam, Domont, France). A standard curve of 1, 1, 3, 3-tetraethoxypropane (TEP) (T9889, 97%, Sigma, St. Louis, MO 63101, USA) as MDA precursor, and at a range of 0–50 µm/g was used to equate the absorbance values and expressed as malondialdehyde (MDA) per g of sample.

### 2.7. Statistical Analysis

This study was subjected to a completely randomized design with all the data analyses were run using the Statistical Analysis System (SAS) 9.4 Version (SAS Institute, Cary, NC, USA). The statistical model used is: Y_ij_ = *µ* + T_i_ + e_ij._, where Y_ij_ is the mean of the jth observation of the ith treatment; *µ* is the sample mean; T_i_ is the effect of the ith treatment, and e_ij_ is the effect of the error. The data were analyzed by the General Linear Model (GLM) procedure of SAS and Duncan Multiple Range Test was used to separate means. Also, an F test was performed for egg yolk coloration to determine the orthogonal contrasts among treatments and to estimate the linear effect of selenium sources. The significance of the statistical difference between the treatments was established at *p*-value < 0.05 level. The assumption of normality was by using the visual assessment of histogram distribution and Quantile-Quantile (Q-Q) plots of model residual. The results were presented as mean ± SEM in all figures and tables.

## 3. Results

### 3.1. Egg Yolk Color

The results of egg yolk color of the initial, last, and 14-days post-stored at 4 ± 2 °C are presented in Table 2. Dietary Se supplementation had an impact (*p* < 0.05) on the color scores determined by the Yolk Color Fan^®^ scale (RYCF). For fresh and stored egg yolk, a significant (*p* < 0.05) difference was observed in the group supplemented with organic Se source, particularly ADS18 when compared to inorganic and unsupplemented hens. Similarly, dietary treatments influenced (*p* < 0.05) the color of egg yolk as measured by the Konica Minolta Chroma Meter CR-410 (CR-410, Konica Minolta, Osaka, Japan) (Table 2). Fresh and post-stored lightness L* were higher (*p* < 0.05) in hens fed a basal diet and an inorganic Se source. The egg yolks of the control and supplemented with inorganic hens were found to be extremely light. The redness (a*) of the egg yolk color was unaffected by the basal or the Se supplemented diets, while organic (Se-Yeast or ADS18) Se-fed hens had a slightly higher redness (a*). All the treatment groups had the same redness means value (a*), which represents the poor red hue values. In terms of yellowness (b*), dietary organic Se supplementation increased (*p* < 0.05) the yellowness of egg yolks (b*) significantly for both fresh and stored eggs. With Chroma (C*), a similar pattern was observed among the treatment groups. Dietary Se supplementation did not affect (*p* > 0.05) Hue (H*) index.

### 3.2. Egg Yolk and Breast Tissue Antioxidant Profile

#### 3.2.1. Total Carotene Content in Egg Yolk, Breast Tissue, and Feed Samples

The total carotenoid in the egg yolk, breast meat, and feed samples is summarized in Table 3. Dietary treatments affected (*p* < 0.05) fresh, stored egg yolk, breast meat, and feed samples. The results showed that hens-fed organic Se sources (ADS18 > Se-Yeast) had higher (*p* < 0.05) carotene content than inorganic and control egg yolk for fresh and post-stored eggs after 14 days. Nonetheless, hen-fed Se-yeast has lower carotene content in their breast muscle than hens fed the basal diet. In general, hens fed an organic bacterial protein-rich diet had higher carotene contents in their egg yolks and breast tissue as compared to other treatment groups. The carotenoid content of stored egg yolk decreases over time (14 days at 4 °C) as compared to fresh egg yolk.

#### 3.2.2. Total Cholesterol Content in Egg Yolk and Breast Tissue

The total cholesterol content of the initial, fresh, stored and breast muscle was presented in Table 3. When fresh, stored egg yolk, and breast muscle were compared to control, total cholesterol level was significantly (*p* < 0.05) lower. For fresh egg yolk, the ADS18, Se-yeast, Na_2_SeO_3_-supplemented groups had the lowest (*p* < 0.05) level of total cholesterol as compared to the control group. In ADS18-fed hens, stored egg yolks with the lowest (*p* < 0.05) total cholesterol concentration showed a similar pattern. Only organic Se (ADS18 > Se-Yeast) supplemented hens had lower (*p* < 0.05) cholesterol than inorganic and control in the breast muscle. However, no dietary Se effect was found in the initial egg yolks because the eggs were collected and examined at the start of the study (3 days).

#### 3.2.3. Total Phenolic Content in Egg Yolk and Breast Tissue

The concentration of total phenols in fresh, stored egg yolk, and chicken breast muscle increased (*p* < 0.05) after dietary Se supplementation (Table 4). The total phenol content of fresh and stored egg yolks was significantly (*p* < 0.05) increased when organic Se was supplemented. Although the fresh egg yolk of inorganic-fed hens did not differ (*p* > 0.05) significantly from that of organic hens, it did differ (*p* < 0.05) significantly from the ADS18 group. Organic Se supplementation (ADS18 or Se-Yeast) increases (*p* < 0.05) the total phenolic content of stored egg yolk and breast meat compared to inorganic and basal diet-fed hens.

#### 3.2.4. Total Flavonoid Content in Egg Yolk and Breast Tissue

The total flavonoid content of fresh, stored egg yolk and breast meat was not (*p* > 0.05) affected by the Se dietary treatment (Table 4). Even though flavonoid concentrations were not statistically significant (*p* > 0.05) different, the Se-yeast group had higher values compared to other stored egg yolk groups. At the early phase of the study, hens supplemented with ADS18 had higher numerical values of flavonoid content in fresh egg yolk than the other treatment groups. Also, the control group had lower flavonoid content in breast muscle than supplemented (inorganic or organic) Se-fed hens.

### 3.3. Oxidative Status Determination

The reducing capacity of antioxidants was centered in a single measure as ‘’Total Antioxidant Capacity’’ (TAC). In the present study, egg yolk and breast meat TAC were evaluated based on their reducing capacity by Phosphomolybdenum (PM) and Ferric reducing antioxidant power (FRAP) assay. The PM assay is based on sample reduction of Phosphate-Mo (VI) to Phosphate Mo (V), resulting in bluish-green phosphate or Mo (V) color formation. Meanwhile, the reduction potential of the sample and normal antioxidant is determined by FRAP as higher absorption implies higher reduction potential and vice versa.

#### 3.3.1. Total Antioxidant Capacity Assay

The total antioxidant capacity of initial, fresh, and stored egg yolk and breast meat was determined by the phosphomolybdenum method and expressed as equivalents of ascorbic acid (mg/g of sample) (Table 5). The antioxidant activity of egg yolk from the hens-fed Se-supplemented diet was higher (*p* < 0.05) than that of fresh egg yolk. The antioxidant activity values in egg yolk stored for 14 d were higher (*p* < 0.05) in Se supplemented groups, but the ADS18-fed hens are more superior to basal diet treatment. However, there were no significant (*p* > 0.05) differences in antioxidant activity between the breast meat treatment groups with values slightly lower than egg yolk.

#### 3.3.2. Ferric Reducing Antioxidant Power (FRAP) Assay

The reduction power assay showed no significant (*p* > 0.05) effect of dietary Se supplementation on initial, stored egg yolk and breast meat (Table 5). Fresh egg yolk, however, demonstrated the highest reduction capacity by ADS18 for bacterial organic-fed hens when compared to Se-Yeast, Na_2_SeO_3,_ and the control group.

#### 3.3.3. Thiobarbituric Acid Reactive Substances (TBARs)

The primary oxidation products (MDA) concentrations in egg yolk, tissue, and blood serum were significantly (*p* < 0.05) reduced by the oxidative stability parameters (Table 6). In general, eggs from hens fed dietary Se had lower MDA (*p* < 0.05) concentration in their yolks than eggs from other treatment groups. In particular, MDA levels in dietary organic Se-supplemented chicken eggs were lower (*p* < 0.05) MDA than inorganic Se and control groups. The MDA concentration increased (*p* < 0.05) during storage time and was related to the storage temperature (4 ± 2 °C) compared to the remaining sampling days with the pattern of the results among the treatment groups remains the same. Breast muscle displayed post-mortem ageing periods of 0, 1, and 5 days, while 0 days was observed for thigh tissue. Furthermore, as compared to the control group, Se supplementation (organic) had a major effect on TBARs values, inhibiting the oxidation process in eggs from organic Se-treated groups during the analysis. The lipid oxidation process increases with progressive time of post-stored egg yolk and post-mortem storage at 4 ± 2 °C. On days 1 and 5, MDA concentrations in the breast tissue of hens-fed bacterial organic Se increased significantly (*p* < 0.05) in the treatment groups, although lower values were recorded in the breast tissue of hens fed-bacterial organic Se. Dietary Se affected the chicken thigh muscle, with organic Se (ADS18 or Se-yeast) being more effective in preventing lipid oxidation. In serum blood, hens fed dietary ADS18 had the lowest MDA (*p* < 0.05) concentration, while control hens had the higher MDA concentrations, with comparable MDA concentrations in the inorganic and Se-yeast treatment groups.

## 4. Discussion

Consumers’ preference for egg selection has now shifted from yolk cholesterol content or fatty acid profile to its color [46]. The dietary supplementation of carotene plays a vital role in egg yolk color intensification [36]; pigment (carotene) synthesis in hen eggs is feasible with its supplementation via diet ingredients [47]. Microorganisms like algae, fungi, some bacteria, and plants were reported to synthesize carotene pigments [48]. In the present study, the L* (lightness), a* (red), b* (yellow), chroma (saturation or color intensity), and hue (color tone) of egg yolks were calculated using the RYCF and CIELAB photo-calorimetric determination systems. The findings showed that dietary supplementation with organic selenium increased egg yolk yellowness and decreased yolk brightness while not affect egg yolk redness in all treatment groups and for both the fresh and post-stored eggs. Similarly, hens provided organic Se supplementation had higher yolk color (fresh and stored) values, as calculated by the Yolk Color Fan^®^ (Roche) scale; however, this scale only determines the sequence number of a stripe range. Since there is a lack of research on the effects of Se on egg yolk color traits measured by a Chroma Meter, some literature findings containing antioxidants are used to support or refute these findings. Gouveia et al. [49] found that xanthophylls are ingested by chickens via the intestinal tract, assimilated into triglyceride-rich lipoproteins (chylomicrons), released into the blood (circulatory system), and transported to the yolk [50]. Since the hens were fed the same basal diet except for Se supplementation (inorganic or organic), the increased yolk color in the treated group may be attributed to the accumulation of xanthophylls in the yolk.

The current findings are consistent with previous results [51], who observed a linear increase in egg yolk color score (RYCF) with dietary incorporation of marine algae (*Spirulina platensis*) at 0.1 to 0.2% (6.3–7.6) and 1.5 to 2.5% (10.55–11.66), respectively, compared to the negative control. Similarly, Park et al. [52] observed similar results when hens were fed with dietary marine microalgae (*Schizochytrium*) powder. Studies on Se supplementation (inorganic or organic) on the egg yolk color are lacking. However, a study by Omri et al. [36] found that supplementing 2% tomato and red pepper mixture reduced (*p* < 0.05) the lightness of yolk color. The egg yolk color index was stated to increase from 8.5 to 14.6 when 130 g of dried tomato peel per kg was added to the diet [53]. Arpasova et al. [54] observed that hens fed lower amounts of Se had lighter egg yolks than those fed higher Se. This resulted in a deeper egg yolk color in the organic (ADS18 or Se-Yeast) treated group. On the contrary, Omri et al. [48] reported an increase in egg yolk redness and decreased yellowness with colorimetric determination when evaluating the effects of *Arthrospira platensis* (spirulina) supplementation on laying hens. However, the latter author [55] found that eggs stored at 4 °C for 30 days corresponding to linseeds-fed hens had increased yellowness and decreased redness. Nonetheless, there are few studies on the impact of storage on egg yolk color. The yolk pigmentation stability of omega 3 (ω-3) enriched eggs stored at room temperature (26.5 °C) and refrigeration (7.9 °C) for 35 d was found to be decreased [56]. Fresh and stored egg yolks saturation (C*) displayed a similar trend of significant differences, but there were no dietary effects on hue angle values to either of the treatment groups. The use of Se supplementation (organic) as an antioxidant will help to reduce the use of synthetic pigments as feed additives in diets of laying hens. Therefore, its stability over a defined period of storage needs to be of primary concern to researchers. It is important to fix undesirable changes (be it chemical, enzymatic, or physical) in appearance and color as well as the quality of the nutrients contained in layers diet, as some can damage and lead to pigment losses during storage. In hens fed different Se sources, the contrast analysis showed significant (*p* < 0.05) differences in the brightness and yellowness of the egg yolk (fresh or stored) and pronounce in the basal diet, inorganic and organic (ADS18 or Se-yeast) diets. For the Chroma (C*) index, there were significant differences between the treatments over the trial period, but no difference was observed for the Hue (H*) index.

Carotenoids are lipid-soluble pigments in plants, insects, birds, and aquatic animals that result from the pigment carotene or carotenoids [57]. They function as a color pigmentation of orange, yellow, or sometimes red. Carotenoids in egg yolk are solely dependent on nutrients available in the feed and thus vary by egg types [58]. The antioxidant Se can help improve the color of egg yolks. Organic selenium or vitamin E supplementation, for example, increased egg yolk carotene concentration [59]. The color of the egg yolk is influenced by oxycarotenoids (xanthophyll pigments) resulting from the hen’s diet, which are lost when oxidized [60]. They are connected to lipoproteins, which are transported to the egg yolk of an egg [61]. The yolk color response to antioxidants influences the stability of lipid-soluble carotenoids available in a hen’s diet or body [62]. Furthermore, carotenoid is an antioxidant that acts as feather dye, vitamin A precursor, and other related endocrine and immune-related functions in poultry [63]. Total carotenoids were measured in this study. As a result, in addition to age-related macular degeneration, the two major egg carotenoids (lutein and zeaxanthin) play an important role in eye macular disease by protecting against light-induced oxidative damage as their mechanism of action [64]. They can attract blue light until it has a passive antioxidant effect on photoreceptor cells [64]. Owing to their possession of double bonds, they have ability to produce a highly resonance-stabilized C-centered radical that help to scavenge hydroxyl and superoxide radicals [65]. Many of these effects are linked to its biological antioxidant function [66]. In this study, the organic bacterial protein (ADS18) had a higher total carotenoid content than the inorganic and basal diet groups. This is consistent with previous findings by Karadas et al. [67], who found a 22-fold increase in carotenoid content in hens supplemented with carotenoids in eggs during pre-and post-hatch studies compare to controls. In vivo studies using dried tomato peel revealed a 2.7-fold increase in β-carotene compared to 1.7 µg/gDM in comparison [68]. There is a scarcity of information on the impact of storage in hens supplemented with similar treatment to that used in the current research. Similar to the current results, eggs stored at room temperature (26.5 °C) or under refrigeration (7.9 °C) for 35 d had lower total carotenoids egg yolk concentrations (28.55 against 22.09 µg/g) and (28.55 against 23.57 µg/g) [56]. In contrast, eggs stored at 2 °C for 56 d [69] and 4 °C for 28 d had no decrease in total carotenoid contents in the egg yolk [36].

Total egg yolk and breast meat cholesterol were significantly decreased by dietary Se supplementation. Se supplementation was reported by Poirier et al. [70] to reduce plasma lipids concentrations of total cholesterol, LDL-cholesterol, and VLDL-cholesterol in male Syrian hamsters. Selenium plays a vital role in the hormonal (thyroid) balance of fat metabolism [71], and its deficiency has been linked to increased 3-hydroxy 3-methylgluatryl CoA (HMG-CoA) reductase activity in liver microsomes [72]. As an antioxidant form of the active center of GPX, Se may help lower cholesterol levels [73]. In their review, Brown and Jessup [74] observed that as the antioxidant level increases in the diet, the cholesterol concentration decreases, and vice versa. Organic (ADS18 > Se-Yeast) Se supplementation, relative to inorganic (Na_2_SeO_3_) and basal diet fed hens, substantially decreased total egg yolk and breast meat cholesterol. A linear reduction in egg yolk and serum cholesterol levels was recorded with an increase of 0, 5, 10, and 15g MPM/Kg in *Moringa oleifera* pod (Lam.) meals [75]. Supplementing with organic Se and vitamin E has also been shown to lower cholesterol levels in serum and egg yolks [24]. Radwan et al. [73] found that nano-Se supplementation reduces total plasma and yolk cholesterol levels (153 mg/dl and 14.0 mg/g) at 0.25 ppm, respectively. Similarly, Attia et al. [76] and Łukaszewicz et al. [77] found a significant reduction in plasma cholesterol at 0.25 ppm and 0.3 mg/kg, of dual-purpose breeding hens of Gimmizah and Japanese quails yolk fed-organic selenium. The lower cholesterol observed in fresh, stored egg yolk and refrigerated breast meat may be attributable to differences in cholesterol synthesis control enzymes in chickens [78]. The reason for cholesterol decrease may be due to the inhibition of sterol biosynthesis by oxysterols. Se supplementation has been shown to increase 15d-PGJ_2_ (15-deoxy-Δ-12, 14 prostaglandin J2) production in response to oxidative stress-induced cell protection [79], a known peroxisome proliferator-activated receptor-γ ligand (PPAR γ) [80]. The activation of the latter by troglitazone regulates the concentration of sterol regulatory element-binding protein (SREBP)-2, resulting in a decreased cholesterol synthesis [81].

The egg white and yolk of a chicken egg contain a lot of antioxidant-rich compounds [9]. Antioxidants are abundant in egg proteins (ovalbumin, ovotransferrin, phosvitin), egg lipids (phospholipids), and micronutrients (vitamin E and A, selenium, and carotenoids) [9]. The type of flavonoids and phenolic acid that play a role in good antioxidant activity was the bioactive compounds in the egg, particularly the albumen [82]. Free radicals can be effectively counteracted by the existence and activity of phenolic acids and flavonoids in the system [82]. Dietary supplementation with Se increased the bioactive (phenolic) content of fresh, stored egg yolk and breast meat in the current research. Phenolics are a major phytochemical class that includes chemical compounds of one or more phenolic groups [83]. The resulting concentration of antioxidants depends on the phenol group and the double bond, i.e., the lower the concentration of antioxidants, the higher its activity [84]. In contrast, Siger et al. [85] found the binding ability to scavenge peroxyl radicals was unconnected with the flavonoid concentration because of the chances of the formation of the phenoxyl radicals. Simple phenolic acids are not easily deposited into chicken egg yolk due to their hydrophilic nature under natural conditions [86]. Since work is scarce in this regard, comparing the current results to other literature studies is difficult. Untea et al. [87] recently reported a significant increase in the total polyphenol content of egg yolk with a 0.5% and 1.0% dietary inclusion of bilberry and walnut leaves, respectively. From hens-fed grounded mixtures of 4.5 and 2% of linseeds and fenugreek seeds, a significant increase in total phenol concentration in yolk was observed compared to 4.5% ground linseeds and 4.5% ground linseeds and 1% each of dried tomato and sweet pepper powder, respectively [53]. A phyto-additives (dried tomato peel) trial on the yolk carotenoids and phenols of laying hens revealed an increase in total phenol content, which was linked to cholesterol reduction [35]. However, the inclusion of varying levels of dietary fennel seed did not have a significant effect on the total phenol content of egg yolk from *Coturnix coturnix japonica* [40]. Gasecka et al. [88] found that Se supplementation at concentrations ranging from 0.5 to 5.0 mmol/L increased the biosynthesis of phenolics and flavonoids in mushrooms. Se treatment also increases the phenolic content of green tea [89], and purple potatoes [90] with the mechanism still unknown. One potential mechanism might be that Se promotes the accumulation of some sugars, such as glucose, which is a crucial substrate in many metabolic pathways [90]. Sae-Lee et al. [89] suggest that Se and Al influenced the content of secondary metabolites such as phenolic substances, but the biosynthesis of these substances is uncertain. Another explanation may be that phenolics and flavonoids have been confirmed to be responsible for antioxidant activity [9,91,92,93], which could be connected to the improved antioxidant enzyme status and higher selenoproteins gene expression [94]. To our knowledge, there is no evidence or literature on total phenol content in response to selenium supplementation in egg yolks and breast meat.

Flavonoids are forms of antioxidants that are water-soluble and have glucose groups in the side chain [95]. The subsequent concentration of antioxidants depends on the phenol group and the double bond presence [84]. However, owing to the possibility of phenoxyl radicals formation, Siger et al. [85] found that the capacity to scavenge peroxyl radicals was independent of flavonoid concentration. The flavonoid content of breast meat and fresh and stored egg yolk showed that selenium supplementation did not affect this parameter. In this respect, literature is scarce, making it difficult to compare our findings. Omri et al. [36] found no differences in flavonoids content of chicken egg yolk supplemented with linseed alone or combined with dried tomato-red pepper mixture before and after storage. Omri and Abdouli [53], however, found that hens supplemented with sweet pepper and dried tomato and fenugreek seeds had higher flavonoid concentrations in egg yolk (1.53 to 2.96 mg CAE/g) and (1.53 to 3.02 mg CAE/g). There are few studies on the impact of selenium supplementation on the total flavonoid content of fresh and stored egg yolks and breast meat.

Eggs are considered an excellent source of dietary antioxidants [96]. In this study, dietary Se supplementation influenced the antioxidant activity of fresh and stored eggs as measured by the reduction of Mo (VI) to Mo(V). Whereas only stored eggs were affected by dietary treatments for ferric reducing power activity. These results suggest that dietary addition of Se to layers diet increased chicken eggs antioxidant capacity. There is no research on egg antioxidant activity expressed as AAE or GAE per g in laying hens in the selenium supplementation literature. Wang et al. [97] recorded higher total antioxidant capacity in eggs from epigallocatechin-3-gallate (EGCG)-fed layers, which is consistent with the present findings. Omri, et al. [36] also found an improvement in antioxidant activity measured by phosphomolybdenum reduction of a mixture of a diet supplemented with ground linseed (4.5%), dried tomato paste (1%), and sweet pepper powder (1%), stored and slightly in the fresh egg for hens-fed (1%). No significant antioxidant activity of egg yolk or meat was found when measured in the above study with a ferric reduction antioxidant power assay. Similarly, egg yolk total antioxidant activity was positively affected in golden pheasants (*Chrysolophus pictus*) fed diets containing various levels of green vegetables [98]. Organic Se was found to increase the carotenoid content while decreasing cholesterol content in egg yolks in the current study, which may have led to the higher egg antioxidant capacity. It is, therefore, logical to speculate that organic Se supplementation (ADS18 or Se-Yeast) would enhance egg yolk antioxidant capacity, because organic Se prevents carotene oxidation, potentially increasing its deposition. McGraw et al. [99] stated an increase in egg antioxidant status during hatching and fleeing in goldfinches may be beneficial. However, further research is needed to explain and understand the underlying mechanisms of this response.

The freshness of eggs is one of the consistency parameters affected by storage time, temperature, and relative humidity [73]. In cells, free radicals can produce reactive substances, which in turn damages cells and tissues. Antioxidants may prevent the damage caused by oxidation. Oxidation intensity of lipids is one of the parameters used as an indicator to assess the freshness of poultry products. MDA is one of the lipid peroxide metabolic products and negatively correlates with the activity of GPX [100]. The degree of peroxidation of fatty acids (animal products) can be monitored by malondialdehyde (MDA) concentrations, i.e., the higher the MDA concentration and thus the degree of lipid peroxidation. A decrease of the content of MDA observed in egg yolk, breast muscle, thigh, and serum may be due to the increase in the activity of GPX resulting from supplemental dietary form (organic vs inorganic). The advantageous effects of organic Se in layers are connected to its efficacy of being transferred to the egg [101]. Organic Se was found to improve the oxidative stability of eggs [24] by reducing the eggshell or fluid’s cellular damage. Generally, due to its antioxidant properties, Se provides fat and protein oxidation stability in the eggs of laying hens fed a dietary Se diet [22]. A study to investigate the interaction between different Se sources and trace elements about the antioxidant system of laying hens is consistent with the present results [102]. Egg-laying hens receiving selenomethionine in stored eggs showed decreased lipid peroxidation, probably increasing the shell life of the eggs [103]. Selenium supplementation at 0.25 ppm showed a significant decrease in MDA content in fresh and stored egg yolk compared with 0.10 ppm supplemented egg yolk [73]. Wang et al. [104] reported a significant increase in GPX activity and decrease yolk MDA content when Se to Langshan layer hens was supplemented with 0.3 mg/kg. More egg freshness was observed by the latter author and Gajčević et al. [105] after a month of storage at 4 °C with 0.4 mg/kg of organic Se supplementation.

The noted increase in MDA content in stored eggs could be attributed to the storage temperature (4 ± 2 °C). Organic Se-supplemented egg yolks, on the other hand, had lower MDA values than inorganic Se and unsupplemented egg yolks. Cimrin et al. [13] recently recorded lower yolk TBARS values in vitamin E-fed hens eggs at room temperature, although refrigerated eggs showed no dietary effects. In Hy-Line W-36 hens trials, Susceptibility to lipid peroxidation and egg yolk decreased with a combination of increased Se and vitamin E concentrations [19]. As a result, the authors predicted that advanced storage would increase MDA concentrations [106]. Skřivan et al. [17] and Asadi et al. [25] found significantly increased yolk lipid peroxidation and MDA content when eggs were stored at 20 °C and refrigerated for 7–14 days. On the contrary, the lipid oxidation stability of fresh egg yolks was not improved by dietary inclusion of linseed mixture, dried tomato paste, and sweet red pepper [36]. The malondialdehyde content of refrigerated egg yolks did not improve after six weeks of storage, according to Nimalaratne et al. [96]. Nadia et al. [107] found that there was a significant difference between the treatments with dietary natural antioxidants but not with storage time.

Though hens (layers) are bred to produce eggs, the quality of their meat is important to ensure oxidative stability after supplementing antioxidants to their diets. As a result, measuring lipid peroxidation using MDA content in breast and thigh muscle can be helpful. Poultry meat, due to its high polyunsaturated fatty acid content, is typically susceptible to rapid deterioration. Ahmad et al. [100] observed significantly reduced lipid peroxidation in fed-selenium yeast chicken breast meat, which is consistent with the current findings. A significant decrease in malondialdehyde (MDA) concentration of 0.15 mg organic Se/kg each in broiler muscle (L-Se-Met or D-Se-Met) compared to inorganic sodium selenite group [108]. In contrast, dietary supplementation with Se did not affect the concentration of MDA (expressed as TBARs) in lamb muscle over 9 days of storage [109]. The activity of GPX in serum and liver, as well as free radical inhibition, is found to have a significant effect to reduce the content of MDA in broiler blood-fed 0.30 mg/kg of nano-Se [110]. However, dietary supplementation with Se did not affect serum MDA concentration [108].

Lipid peroxidation is a complex pathway in which fatty acyl hydroperoxides form as a free radical chain in the reaction process between unsaturated fatty acids and reactive oxygen species [100]. Lipid degradation and oxidative rancidity are caused by a sequence of secondary reactions that follow primary autoxidation and result in changes in flavor, nutrient quality loss, and environmental pollution, among others [111]. Organic selenium (ADS18 > Se-Yeast) supplementation reduced MDA concentration in egg yolks, breast and thigh tissue, and blood throughout the study. The difference in responses between different sources of Se may be attributable to differences in metabolic pathways (inorganic or organic), as organic sources preserved the integrity of muscle cells linked to lipid oxidation and oxidative stability [112].

## 5. Conclusions

The current findings show that dietary supplementation with Se, especially organic (ADS18 > Se-Yeast) forms, improved egg yolk color, antioxidant profile, and oxidative status in laying hen egg yolks and tissues. Furthermore, organic selenium supplementation strengthened the antioxidant profile of egg yolk and tissue (increased total carotenoid and phenol content) and oxidative stability (reduced cholesterol). It is worth noting that organic Se-rich eggs can be kept fresh for up to two weeks. The source of supplemented organic Se is essential for egg enrichment and antioxidant properties. Thus, it is possible to produce ‘’functional eggs or meat’’ enriched with organic selenium.

## Figures and Tables

**Table 1:** Ingredient compositions and analyzed nutrient levels of the basal diet (on dry matter basis).

Ingredients	Layers
Corn	44.00
Soybean Meal 48%	29.00
Wheat Pollard	11.00
CPO	3.50
L-Lysine	0.10
DL-Methionine	0.25
Dicalcium Phosphate (18%)	2.00
Calcium Carbonate	7.70
Choline Chloride	0.10
Salt	0.35
Mineral Mix *	0.60
Vitamin Mix **	0.60
Antioxidant ***	0.40
Toxin Binder ****	0.40
**Total**	**100**
Analyzed Composition	
Metabolizable energy Kcal/Kg	2761.24
Crude protein (%)	17.66
Fat (%)	5.3
Fiber (%)	3.98
Calcium (%)	3.65
Total Phosphorus (%)	0.88
Av. Phosphorus for poultry (%)	0.48

* Mineral premix supplied (per kg of diet): copper 15 mg, zinc 120 mg, iron 120 mg, manganese 150 mg, iodine 1.5 mg, and cobalt 0.4 mg. ** Vitamin premix supplied (per kg of diet): Vitamin A (retinyl acetate) 10.32 mg, vitamin E (DL-tocopherol acetate) 90 mg, cholecalciferol 0.250 mg, vitamin K 6 mg, cobalamin 0.07 mg, thiamine 7 mg, riboflavin 22 mg, niacin 120 mg, folic acid 3 mg, biotin 0.04 mg, pantothenic acid 35 mg and pyridoxine 12 mg. *** Antioxidant contains butylated hydroxyanisole (BHA). **** Toxin binder contains natural hydrated sodium calcium aluminum silicates to reduce the exposure of feed to mycotoxins. Feed live International Software (Nonthaburi, Thailand) was used to formulate the diets.

**Table 2:** Egg yolk coloration before and after storage at 4 ± 2 °C for 14 days of laying hens supplemented with sodium selenite, selenium yeast, and bacterial organic source.

Parameters		Dietary Treatments ^1^				*p*-Value	Contrast, *p*-Value		
	Eggs	Con	Na_2_SeO_3_	Se-Yeast	ADS18		Unsupplemented vs. Supplemented	Inorganic vs. Organic	Se-Yeast vs. ADS18
RYCF	Initial	1.83 ± 0.30	1.83 ± 0.24	2.50 ± 0.29	2.00 ± 0.21	0.2422	0.3641	0.2014	0.1846
	Fresh	2.83 ± 0.30 ^b^	2.92 ± 0.26 ^b^	3.25 ± 0.18 ^a,b^	3.67 ± 0.14 ^a^	0.0536	0.0988	0.0591	0.2034
	Stored	2.92 ± 0.26 ^b^	2.92 ± 0.36 ^b^	3.33 ± 0.26 ^b^	4.17 ± 0.21 ^a^	0.0072	0.0881	0.0176	0.0382
L*	Initial	57.91 ± 0.33	56.26 ± 0.70	55.78 ± 0.83	56.25 ± 1.07	0.2414	0.0482	0.7916	0.6786
	Fresh	70.79 ± 0.44 ^a^	72.22 ± 0.54 ^a^	65.05 ± 0.90 ^b^	62.83 ± 0.54 ^c^	<0.0001	<0.0001	<0.0001	0.0154
	Stored	71.22 ± 0.35 ^a^	68.19 ± 0.66 ^b^	66.91 ± 0.23 ^b,c^	65.43 ± 0.85 ^c^	<0.0001	<0.0001	0.0055	0.0721
a*	Initial	1.83 ± 0.11	1.55 ± 0.15	1.75 ± 0.21	1.80 ± 0.18	0.6228	0.5023	0.2628	0.8347
	Fresh	1.11 ± 0.08	1.12 ± 0.14	0.93 ± 0.11	1.09 ± 0.12	0.5954	0.6273	0.436	0.3088
	Stored	0.47 ± 0.06	0.48 ± 0.05	0.55 ± 0.05	0.55 ± 0.06	0.596	0.358	0.3113	0.954
b*	Initial	31.87 ± 0.34	31.83 ± 0.31	32.17 ± 0.19	32.34 ± 0.26	0.5212	0.4614	0.2195	0.6634
	Fresh	45.85 ± 0.30 ^c^	46.59 ± 0.36 ^b,c^	47.24 ± 0.24 ^b^	49.76 ± 0.42 ^a^	<0.0001	<0.0001	<0.0001	<0.0001
	Stored	47.63 ± 0.44 ^d^	49.15 ± 0.54 ^c^	50.68 ± 0.36 ^b^	52.26 ± 0.34 ^a^	<0.0001	<0.0001	<0.0001	0.011
C*	Initial	31.93 ± 0.33	31.87 ± 0.32	32.23 ± 0.19	32.40 ± 0.27	0.5093	0.4667	0.2061	0.6702
	Fresh	45.87 ± 0.30 ^c^	46.60 ± 0.36 ^b,c^	47.26 ± 0.24 ^b^	49.78 ± 0.42 ^a^	<0.0001	<0.0001	<0.0001	<0.0001
	Stored	47.63 ± 0.44 ^d^	49.15 ± 0.54 ^c^	50.68 ± 0.36 ^b^	52.26 ± 0.34 ^a^	<0.0001	<0.0001	<0.0001	0.011
H*	Initial	1.51 ± 0.004	1.52 ± 0.005	1.52 ± 0.006	1.52 ± 0.005	0.6022	0.3844	0.3015	0.8866
	Fresh	1.55 ± 0.002	1.55 ± 0.003	1.55 ± 0.002	1.55 ± 0.002	0.5029	0.4224	0.2704	0.4871
	Stored	1.56 ± 0.001	1.56 ± 0.001	1.56 ± 0.001	1.56 ± 0.001	0.8445	0.6146	0.473	0.8356

^1^ Con = Control, Na_2_SeO_3_ = Sodium selenite; Se-yeast = Selenium yeast; ADS18 = *Stenotrophomonas maltophilia*, ^a–c^ Mean in the same row with different superscripts are significantly different (*p* < 0.05); RYCF: Roche Yolk Color Fan; L*: lightness, a*: redness, b*: yellowness, c*: chroma and h*: hue angle.

**Table 3:** Total carotenoid (mg/g of β-carotene) and total cholesterol (mg/g) content pre- and post-storage at 4 ± 2 °C for 2 weeks of laying hens supplemented with inorganic selenium and different organic Se sources.

Parameters	Days	Dietary Treatments ^1^				*p*-Value
		Con	Na_2_SeO_3_	Se-Yeast	ADS18	
Total Carotene						
Egg yolk	Initial	21.23 ± 0.58	21.80 ± 0.35	22.51 ± 0.41	21.21 ± 0.39	0.1597
	Fresh	20.49 ± 0.61 ^b,c^	20.10 ± 1.12 ^c^	22.15 ± 0.32 ^a,b^	23.37 ± 0.37 ^a^	0.0086
	Stored	15.77 ± 1.20 ^b^	17.95 ± 0.56 ^b^	20.97 ± 0.82 ^a^	22.13 ± 0.49 ^a^	<0.0001
Breast meat	NA	2.07 ± 0.06 ^a^	1.99 ± 0.05 ^a,b^	1.88 ± 0.02 ^b^	1.95 ± 0.03 ^a,b^	0.0267
Feed sample	NA	5.14 ± 0.16 ^b^	5.16 ± 0.21 ^b^	5.59 ± 0.08 ^b^	6.27 ± 0.14 ^a^	0.0001
Total Cholesterol						
Egg yolk	Initial	12.75 ± 0.24	12.75 ± 0.43	13.25 ± 0.37	12.94 ± 0.33	0.7076
	Fresh	27.83 ± 0.44 ^a^	25.70 ± 0.41 ^b^	22.03 ± 0.51 ^c^	20.12 ± 0.48 ^d^	<0.0001
	Stored	27.92 ± 0.45 ^a^	26.35 ± 0.60 ^a,b^	24.78 ± 0.54 ^b^	19.83 ± 0.91 ^c^	<0.0001
Breast meat	NA	16.17 ± 0.40 ^a^	15.20 ± 0.18 ^a^	12.33 ± 0.44 ^b^	9.09 ± 0.36 ^c^	<0.0001

^1^ Con = control, Na_2_SeO_3_ = Sodium selenite; Se-yeast = Selenium yeast; ADS18 = *Stenotrophomonas maltophilia*. Initial = day 3, fresh = day 95 and stored = day 109 for 2 weeks. ^a–d^ Mean in the same row with different superscripts are significantly different (*p* < 0.05). NA = not applicable.

**Table 4:** Pre- and post-storage egg yolk and breast muscle 4 ± 2 °C for 14 days.

Parameters	Days	Dietary Treatments ^1^				*p*-Value
		Con	Na_2_SeO_3_	Se-Yeast	ADS18	
Total Phenol, mg GAE/g *						
Egg yolk	Initial	2.63 ± 0.037	2.71 ± 0.071	2.77 ± 0.067	2.81 ± 0.047	0.1873
	Fresh	1.47 ± 0.20 ^c^	2.38 ± 0.19 ^b^	2.56 ± 0.11 ^a,b^	2.90 ± 0.10 ^a^	0.0002
	Stored	1.52 ± 0.06 ^b^	1.45 ± 0.03 ^b^	1.89 ± 0.08 ^a^	1.96 ± 0.09 ^a^	0.0004
Breast meat	NA	2.16 ± 0.10 ^b^	2.18 ± 0.16 ^b^	2.92 ± 0.29 ^a^	3.41 ± 0.05 ^a^	0.0006
Total Flavonoid, mg RE/g **						
Egg yolk	Initial	2.21 ± 0.52	1.48 ± 0.16	1.73 ± 0.36	2.28 ± 0.18	0.3293
	Fresh	1.45 ± 0.46	1.73 ± 0.27	1.50 ± 0.21	1.38 ± 0.53	0.9245
	Stored	1.79 ± 0.07	1.68 ± 0.10	1.83 ± 0.10	1.74 ± 0.12	0.7148
Breast meat	NA	1.42 ± 0.32	1.69 ± 0.15	1.69 ± 0.38	1.57 ± 0.36	0.5416

^1^ Con = control, Na_2_SeO_3_ = Sodium selenite; Se-yeast = Selenium yeast; ADS18 = *Stenotrophomonas maltophilia*. Initial = day 3, fresh = day 95 and stored = day 109 for 2 weeks. *: Total phenols expressed in mg gallic acid equivalent, mg GAE/g; **: Flavonoids express in mg rutin equivalent, mg RE/g; ^a–c^ Mean in the same row with different superscripts are significantly different (*p* < 0.05). NA = not applicable.

**Table 5:** Total antioxidant capacity of pre-and post-stored egg yolk and breast tissue for 14 days at 4 ± 2 °C.

Assay	Parameters	Days	Dietary Treatments ^1^				
			Con	Na_2_SeO_3_	Se-Yeast	ADS18	*p*-Value
Phosphomolybdenum Assay (Antioxidant activity, mg AAE/g) *	Egg yolk	Initial	0.82 ± 0.07	0.87 ± 0.04	0.81 ± 0.04	0.86 ± 0.06	0.8073
		Fresh	0.83 ± 0.05 ^d^	1.12 ± 0.02 ^c^	1.40 ± 0.04 ^b^	1.81 ± 0.04 ^a^	<0.0001
		Stored	1.50 ± 0.15 ^c^	1.55 ± 0.11 ^b,c^	1.93 ± 0.16 ^a,b^	2.11 ± 0.06 ^a^	0.0127
	Breast meat	NA	0.77 ± 0.05	0.74 ± 0.08	0.77 ± 0.06	0.73 ± 0.05	0.957
Ferric Reducing Antioxidant Power (FRAP) assay (Antioxidant activity, mg GAE/g) **	Egg yolk	Initial	0.84 ± 0.02	0.88 ± 0.01	0.85 ± 0.01	0.84 ± 0.01	0.2238
		Fresh	1.73 ± 0.07 ^b^	1.78 ± 0.06 ^b^	1.90 ± 0.08 ^b^	2.23 ± 0.04 ^a^	0.0005
		Stored	3.16 ± 0.09	3.01 ± 0.07	3.08 ± 0.05	3.12 ± 0.06	0.4896
	Breast meat	NA	1.90 ± 0.10	2.06 ± 0.13	2.27 ± 0.18	2.13 ± 0.11	0.294

^1^ Con = control, Na_2_SeO_3_ = sodium selenite; Se-yeast = Selenium yeast; ADS18 = *Stenotrophomonas maltophilia*. Initial = day 3, fresh = day 95 and stored = day 109 for 2 weeks. *: Antioxidant activity evaluated as Phosphomolybdenum reducing power and express in ascorbic acid equivalent (AAE). **: Antioxidant activity evaluated as ferric reducing power and expressed gallic acid equivalent (GAE); ^a–d^ Mean in the same row with different superscripts are significantly different (*p* < 0.05). NA = not applicable.

**Table 6:** Effects of different Se sources on oxidative stability of pre-and post-stored egg yolk, breast, and thigh muscle.

Parameters	Days	Dietary Treatments ^1^				*p*-Value
		Con	Na_2_SeO_3_	Se-Yeast	ADS18	
Egg yolks, µg MDA/Kg	D 3	0.093 ± 0.03	0.092 ± 0.032	0.084 ± 0.006	0.088 ± 0.004	0.4105
	D 46	0.129 ± 0.004 ^a^	0.117 ± 0.002 ^a^	0.102 ± 0.004 ^b^	0.082 ± 0.006 ^c^	<0.0001
	D 60	0.133 ± 0.007 ^a^	0.111 ± 0.007 ^b^	0.092 ± 0.004 ^c^	0.084 ± 0.004 ^c^	<0.0001
	D 74	0.118 ± 0.004 ^a^	0.109 ± 0.002 ^b^	0.102 ± 0.001 ^c^	0.096 ± 0.002 ^c^	<0.0001
	D 95	0.114 ± 0.004 ^a^	0.104 ± 0.003 ^a^	0.086 ± 0.003 ^b^	0.077 ± 0.003 ^b^	<0.0001
	D 109 *	0.148 ± 0.010 ^a^	0.127 ± 0.005 ^b^	0.105 ± 0.004 ^c^	0.084 ± 0.002 ^d^	<0.0001
Breast meat, µg MDA/g	D 0	11.46 ± 0.51 ^a^	10.05 ± 0.40 ^b^	9.85 ± 0.09 ^b^	8.72 ± 0.10 ^c^	0.0001
	D 1	10.69 ± 1.40 ^a,b^	12.15 ± 1.17 ^a^	8.23 ± 0.92 ^b^	7.59 ± 0.55 ^b^	0.0211
	D 5	17.79 ± 1.60 ^a^	16.31 ± 1.17 ^a,b^	15.64 ± 0.74 ^a,b^	13.62 ± 0.53 ^b^	0.0854
Thigh, µg MDA/g	NA	27.00 ± 0.89 ^a^	26.39 ± 1.38 ^a^	22.62 ± 1.21 ^b^	18.95 ± 0.84 ^c^	0.0001
Serum, nmol MDA/mL	NA	0.184 ± 0.006 ^a^	0.179 ± 0.006 ^a b^	0.169 ± 0.002 ^b c^	0.159 ± 0.003 ^c^	0.0046

^1^ Con = control, Na_2_SeO_3_ = sodium selenite; Se-yeast = Selenium yeast; ADS18 = *Stenotrophomonas maltophilia*. Initial = day 3, fresh = day 95 and stored = day 109 for 2 weeks. *: D109; Eggs were stored for 14 days at 4 ± 2 °C prior to analysis ^a–d^ Mean in the same row with different superscripts are significantly different (*p* < 0.05). NA = not applicable.

## References

[1] Stadelman W.J., Cotterill O.J., Stadelman W.J., Cotterill O.J. (2001). Egg Science and Technology.

[2] Fisinin V.I., Papazyan T.T., Surai P.F. (2009). Producing selenium-enriched eggs and meat to improve the selenium status of the general population. Crit. Rev. Biotechnol..

[3] Li-Chan E.C., Kim H.O., Mine Y. (2008). Structure and chemical composition of eggs. Egg Bioscience and Biotechnology.

[4] Attia Y.A., Al-Harthi M.A., Korish M.A., Shiboob M.M. (2015). Fatty acid and cholesterol profiles and hypocholesterolemic, atherogenic, and thrombogenic indices of table eggs in the retail market. Lipids Health Dis..

[5] Attia Y.A., Al-Harthi M.A., Shiboob M.M. (2014). Evaluation of quality and nutrient contents of table eggs from different sources in the retail market. Ital. J. Anim. Sci..

[6] Elkin R.G. (2006). Reducing shell egg cholesterol content. I. Overview, genetic approaches, and nutritional strategies. World’s Poult. Sci. J..

[7] Weggemans R.M., Zock P.L., Katan M.B. (2001). Dietary cholesterol from eggs increases the ratio of total cholesterol to high-density lipoprotein cholesterol in humans: A meta-analysis. Am. J. Clin. Nutr..

[8] Lee A., Griffin B. (2006). Dietary cholesterol, eggs and coronary heart disease risk in perspective. Nutr. Bull..

[9] Nimalaratne C., Wu J. (2015). Hen egg as an antioxidant food commodity: A review. Nutrients.

[10] Zdrojewicz Z., Herman M., Starostecka E. (2016). Hen’s egg as a source of valuable biologically active substances. Postepy Hig. Med. Dosw..

[11] Wang J., Liao W., Nimalaratne C., Chakrabarti S., Wu J. (2018). Purification and characterization of antioxidant peptides from cooked eggs using a dynamic in vitro gastrointestinal model in vascular smooth muscle A7r5 cells. NPJ Sci. Food.

[12] Santini A., Novellino E. (2018). Nutraceuticals—shedding light on the grey area between pharmaceuticals and food. Expert Rev. Clin. Pharmacol..

[13] Cimrin T., Avsaroglu M.D., Tunca R., Ivgin K.S., Ayasan T. (2019). Diets with natural and synthetic antioxidant additives on yolk lipid peroxidation and fatty acid composition of eggs stored at different temperatures and duration. Braz. J. Food Technol..

[14] Tapiero H., Townsend D.M., Tew K.D. (2003). The antioxidant role of selenium and seleno-compounds. Biomed. Pharmacother..

[15] Heindl J., Ledvinka Z., Tůmová E., Zita L. (2010). Review the importance, utilization and sources of selenium for poultry: A review. Sci. Agric. Bohem..

[16] Skřivan M., Bubancová I., Marounek M., Dlouhá G. (2010). Selenium and α-tocopherol content in eggs produced by hens that were fed diets supplemented with selenomethionine, sodium selenite and vitamin E. Czech J. Anim. Sci..

[17] Surai P.F., Karadas F., Pappas A.C., Sparks N.H.C. (2006). Effect of organic selenium in quail diet on its accumulation in tissues and transfer to the progeny. Br. Poult. Sci..

[18] Pham-Huy L.A., He H., Pham-Huy C. (2008). Free radicals, antioxidants in disease and health. Int. J. Biomed. Sci..

[19] Mohiti-Asli M., Shariatmadari F., Lotfollahian H., Mazuji M.T. (2008). Effects of supplementing layer hen diets with selenium and vitamin E on egg quality, lipid oxidation and fatty acid composition during storage. Can. J. Anim. Sci..

[20] Superti F., Ammendolia M.G., Berlutti F., Valenti P., Rainer H., Rosina L., Marc A., Rüdiger S. (2007). Ovotransferrin. Bioactive Egg Compounds.

[21] Wang Q., Jin G., Wang N., Guo X., Jin Y., Ma M. (2017). Lipolysis and oxidation of lipids during egg storage at different temperatures. Czech J. Food Sci..

[22] Fasiangova M., Borilova G. (2017). Impact of Se supplementation on the oxidation stability of eggs. World’s Poult. Sci. J..

[23] Tanguy S., Grauzam S., de Leiris J., Boucher F. (2012). Impact of dietary selenium intake on cardiac health: Experimental approaches and human studies. Mol. Nutr. Food Res..

[24] Asadi F., Shariatmadari F., Karimi-Torshizi M.A., Mohiti-Asli M., Ghanaatparast-Rashti M. (2017). Comparison of different selenium sources and vitamin E in laying hen diet and their influences on egg selenium and cholesterol content, quality and oxidative stability. Iran. J. Appl. Anim. Sci..

[25] Surai P.F., Kochish I.I., Fisinin V.I., Velichko O.A. (2018). Selenium in poultry nutrition: From sodium selenite to organic selenium sources. J. Poult. Sci..

[26] Esmaeili S., Khosravi-darani K. (2014). Selenium-enriched yeast: As selenium source for nutritional purpose. Curr. Nutr. Food Sci..

[27] Dalia A.M., Loh T.C., Sazili A.Q., Jahromi M.F., Samsudin A.A. (2017). Characterization and identification of organic selenium-enriched bacteria isolated from rumen fluid and hot spring water. Microbiol. Biotechnol. Lett..

[28] NRC (1994). Nutrient Requirements of Poultry.

[29] Moreda-Piñeiro J., Moreda-Piñeiro A., Bermejo-Barrera P. (2017). In vivo and in vitro testing for selenium and selenium compounds bioavailability assessment in foodstuff. Crit. Rev. Food Sci. Nutr..

[30] Gammelgaard B., Rasmussen L.H., Gabel-Jensen C., Steffansen B. (2012). Estimating intestinal absorption of inorganic and organic selenium compounds by in vitro flux and biotransformation studies in Caco-2 cells and ICP-MS detection. Biol. Trace Elem. Res..

[31] Lipiec E., Siara G., Bierla K., Ouerdane L., Szpunar J. (2010). Determination of selenomethionine, selenocysteine, and inorganic selenium in eggs by HPLC-inductively coupled plasma mass spectrometry. Anal. Bioanal. Chem..

[32] (2018). Lohmann Brown-Classic Management Guide, Lohmann Tierzucht Lohmann. https://www.ltz.de.

[33] (2009). Malaysian Standard. Halal Food-Production, Preparation, Handling and Storage-General Guidelines (Second Revision). http://www.halal.gov.my/v4/index.php?data=bW9kdWxlcy9uZXdzOzs7Ow==&utama=panduan&ids=gp2.

[34] Humam A.M., Loh T.C., Foo H.L., Izuddin W.I., Zulkifli I., Samsudin A.A., Mustapha N.M. (2021). Supplementation of postbiotic RI11 improves antioxidant enzyme activity, upregulated gut barrier genes, and reduced cytokine, acute phase protein, and heat shock protein 70 gene expression levels in heat-stressed broilers. Poult. Sci..

[35] Benakmoum A., Larid R., Zidani S. (2013). Enriching egg yolk with carotenoids & phenols. Int. J. Biol. Biomol. Agric. Food Biotechnol. Eng..

[36] Omri B., Alloui N., Durazzo A., Lucarini M., Aiello A., Romano R., Santini A., Abdouli H. (2019). Egg yolk antioxidants profiles: Effect of diet supplementation with linseeds and tomato-red pepper mixture before and after storage. Foods.

[37] Bianchi M., Petracci M., Cavani C. (2006). The influence of genotype, market live weight, transportation, and holding conditions prior to slaughter on broiler breast meat color. Poult. Sci..

[38] Okonkwo J.C. (2009). Effects of breed and storage duration on the beta-carotene content of egg yolk. Pak. J. Nutr..

[39] Rudel L.L., Morris M.D. (1973). Determination of cholesterol using o-phthalaldehyde. J. Lipid Res..

[40] Buğdayci K.E., Oğuz F.K., Oğuz M.N., Kuter E. (2018). Effects of fennel seed supplementation of ration on performance, egg quality, serum cholesterol, and total phenol content of egg yolk of laying quails. Rev. Bras. Zootec..

[41] Kamtekar S., Keer V., Patil V. (2014). Estimation of phenolic content, flavonoid content, antioxidant and alpha amylase inhibitory activity of marketed polyherbal formulation. J. Appl. Pharm. Sci..

[42] Patel A., Patel A., Patel N.M. (2010). Estimation of flavonoid, polyphenolic content and in-vitro antioxidant capacity of leaves of *Tephrosia purpurea*. Int. J. Pharma Sci. Res..

[43] Benzie I.F.F., Szeto Y.T. (1999). Total antioxidant capacity of teas by the ferric reducing/antioxidant power assay. J. Agric. Food Chem..

[44] Lim Y.Y., Murtijaya J. (2007). Antioxidant properties of *Phyllanthus amarus* extracts as affected by different drying methods. LWT Food Sci. Technol..

[45] Botsoglou N.A., Fletouris D.J., Papageorgiou G.E., Vassilopoulos V.N., Mantis A.J., Trakatellis A.G. (1994). Rapid, sensitive, and specific thiobarbituric acid method for measuring lipid peroxidation in animal tissue, food, and feedstuff samples. J. Agric. Food Chem..

[46] Englmaierová M., Skřivan M., Bubancová I. (2013). A comparison of lutein, spray-dried Chlorella, and synthetic carotenoids effects on yolk color, oxidative stability, and reproductive performance of laying hens. Czech J. Anim. Sci..

[47] Marusich W., De Ritter E., Bauernfeind J.C. (1960). Evaluation of carotenoid pigments for coloring egg yolks. Poult. Sci..

[48] Omri B., Amraoui M., Tarek A., Lucarini M., Durazzo A., Cicero N., Santini A., Kamoun M. (2019). *Arthrospira platensis* (Spirulina) supplementation on laying hens’ performance: Eggs physical, chemical, and sensorial qualities. Foods.

[49] Gouveia L., Veloso V., Reis A., Fernandas H., Novais J., Empis J. (1996). *Chlorella vulgaris* used to color egg yolk. J. Sci. Food Agric..

[50] Salma U., Miah A.G., Tareq K.M.A., Maki T., Tsujii H. (2007). Effect of dietary *Rhodobacter capsulatus* on egg-yolk cholesterol and laying hen performance. Poult. Sci..

[51] Mariey Y.A., Samak H., Ibrahem M. (2012). Effect of using *Spirulina platensis* algae as a feed additive for poultry diets: 1-productive and reproductive performances of local laying hens. Egypt. Poult. Sci..

[52] Park J.H., Upadhaya S.D., Kim I.H. (2015). Effect of dietary marine microalgae (*Schizochytrium*) powder on egg production, blood lipid profiles, egg quality, and fatty acid composition of egg yolk in layers. Asian Australas. J. Anim. Sci..

[53] Omri B., Chalghoumi R., Abdouli H. (2017). Study of the Effects of dietary supplementation of linseeds, fenugreek seeds and tomato-pepper mix on laying hens performances, egg yolk lipids and antioxidants profiles and lipid oxidation status. J. Anim. Sci. Livest. Prod..

[54] Arpášová H., Mellen M., Kačániová M., Haščík P., Petrovič V., Čobanová K., Leng L. (2009). Effects of dietary supplementation of sodium selenite and selenized yeast on selected qualitative parameters of laying hens eggs. Slovak J. Anim. Sci..

[55] Abdouli H., Belhouane S., Hcini E. (2014). Effect of fenugreek seeds on hens’ egg yolk color and sensory quality. J. New Sci..

[56] Barbosa V.C., Gaspar A., Calixto L.F.L., Agostinho T.S.P. (2011). Stability of the pigmentation of egg yolks enriched with omega-3 and carophyll stored at room temperature and under refrigeration. Rev. Bras. Zootec..

[57] Surai P.F., Fisinin V.I., Karadas F. (2016). Antioxidant systems in chick embryo development. Part 1. Vitamin E, carotenoids and selenium. Anim. Nutr..

[58] Karadas F., Grammenidis E., Surai P.F., Acamovic T., Sparks N.H.C. (2006). Effects of carotenoids from lucerne, marigold and tomato on egg yolk pigmentation and carotenoid composition. Br. Poult. Sci..

[59] Surai P.F. (2000). Effect of selenium and vitamin E content of the maternal diet on the antioxidant system of the yolk and the developing chick. Br. Poult. Sci..

[60] Mohiti-Asli M., Shariatmadari F., Lotfollahian H. (2010). The influence of dietary vitamin E and selenium on egg production parameters, serum and yolk cholesterol and antibody response of laying hen exposed to high environmental temperature. Arch. Geflügelk..

[61] Surai P.F., Bortolotti G.R., Fidgett A.L., Blount J.D., Speake B.K. (2001). Effects of piscivory on the fatty acid profiles and antioxidants of avian yolk: Studies on eggs of the gannet, skua, pelican and cormorant. J. Zool..

[62] Surai P.F. (2012). The antioxidant properties of canthaxanthin and its potential effects in the poultry eggs and on embryonic development of the chick. Part 1. World’s Poult. Sci. J..

[63] Møller A.P., Biard C., Blount J.D., Houston D.C., Ninni P., Saino N., Surai P.F. (2000). Carotenoid-dependent Signals: Indicators of foraging efficiency, immunocompetence or detoxification ability?. Avian Poult. Biol. Rev..

[64] Krinsky N.I., Landrum J.T., Bone R.A. (2003). Biologic mechanisms of the protective role of lutein and zeaxanthin in the eye. Annu. Rev. Nutr..

[65] Trevithick-Sutton C.C., Foote C.S., Collins M., Trevithick J.R. (2006). The retinal carotenoids zeaxanthin and lutein scavenge superoxide and hydroxyl radicals: A chemiluminescence and ESR study. Mol. Vis..

[66] Chopra P.A., Willson R.L., Thurnham D.I. (2008). Free radical scavenging of lutein in vitro. Ann. N. Y. Acad. Sci..

[67] Karadas F., Pappas A.C., Surai P.F., Speake B.K. (2005). Embryonic development within carotenoid-enriched eggs influences the post-hatch carotenoid status of the chicken. Comp. Biochem. Physiol. B Biochem. Mol. Biol..

[68] Akdemir F., Orhan C., Sahin N., Sahin K., Hayirli A. (2012). Tomato powder in laying hen diets: Effects on concentrations of yolk carotenoids and lipid peroxidation. Br. Poult. Sci..

[69] Gawecki K., Potkanmski A., Lipinska H. (1977). Effect of carophyll yellow and carophyll red added to comercial feeds for laying hens on yolk color and its stability during short-term refrigeration. Rocz. Akad. Rol. W Pozn..

[70] Poirier J., Cockell K., Hidiroglou N., Madere R., Trick K., Kubow S. (2002). The effects of vitamin E and selenium intake on oxidative stress and plasma lipids in hamsters fed fish oil. Lipids.

[71] Masukawa T., Goto J., Iwata H. (1983). Impaired metabolism of arachidonate in selenium deficient animals. Experrientia.

[72] Nassir F., Moundras C., Bayle D., Sérougne C., Gueux E., Rock E., Rayssiguier Y., Mazur A. (1997). Effect of selenium deficiency on hepatic lipid and lipoprotein metabolism in the rat. Br. J. Nutr..

[73] Radwan N.L., Salah Eldin T.A., El-Zaiat A.A., Mostafa M.A.S.A. (2015). Effect of dietary nano-selenium supplementation on selenium content and oxidative stability in table eggs and productive performance of laying hens. Int. J. Poult. Sci..

[74] Brown A.J., Jessup W. (1999). Oxysterols and atherosclerosis. Atherosclerosis.

[75] Ahmad S., Khalique A., Pasha T.N., Mehmood S., Hussain K., Ahmad S., Shaheen M.S., Naeem M., Shafiq M. (2017). Effect of *Moringa oleifera* (Lam.) pods as feed additive on egg antioxidants, chemical composition and performance of commercial layers. S. Afr. J. Anim. Sci..

[76] Attia Y.A., Abdalah A.A., Zeweil H.S., Bovera F., Tag El-Din A.A., Araft M.A. (2010). Effect of inorganic or organic selenium supplementation on productive performance, egg quality and some physiological traits of dual-purpose breeding hens. Czech J. Anim. Sci..

[77] Łukaszewicz E., Korzeniowska M., Kowalczyk A., Bobak L. (2011). Effect of feed supplementation with organic selenium and vitamin E on freezability of Japanese quail (*Coturnix coturnix*) semen. Pol. J. Food Nutr. Sci..

[78] Konjufca V.H., Pesti G.M., Bakalli R.I. (1997). Modulation of cholesterol levels in broiler meat by dietary garlic and copper. Poult. Sci..

[79] Vunta H., Davis F., Palempalli U.D., Bhat D., Arner R.J., Thompson J.T., Peterson D.G., Reddy C.C., Prabhu K.S. (2007). The anti-inflammatory effects of selenium are mediated through 15-deoxy-Δ12,14-prostaglandin J2 in macrophages. J. Biol. Chem..

[80] Touyz R.M., Schiffrin E.L. (2006). Peroxisome proliferator-activated receptors in vascular biology-molecular mechanisms and clinical implications. Vascul. Pharmacol..

[81] Klopotek A., Hirche F., Eder K. (2006). PPARγ ligand troglitazone lowers cholesterol synthesis in hepg2 and caco-2 cells via a reduced concentration of nuclear SREBP-2. Exp. Biol. Med..

[82] Settharaksa S., Jongjareonrak A., Hmadhlu P., Chansuwan W., Siripongvutikorn S. (2012). Flavonoid, phenolic contents and antioxidant properties of thai hot curry paste extract and its ingredients as affected of pH, solvent types and high temperature. Int. Food Res. J..

[83] Bolling B.W., Chen C.Y.O., McKay D.L., Blumberg J.B. (2011). Tree nut phytochemicals: Composition, antioxidant capacity, bioactivity, impact factors. A systematic review of almonds, Brazils, cashews, hazelnuts, macadamias, pecans, pine nuts, pistachios and walnuts. Nutr. Res. Rev..

[84] Kim H.J., Chang E.J., Cho S.H., Chung S.K., Park H.D., Choi S.W. (2002). Antioxidative activity of resveratrol and its derivatives isolated from seeds of Paeoni. Biosci. Biotechnol. Biochem..

[85] Siger A., Czubinski J., Kachlicki P., Dwiecki K., Lampart-Szczapa E., Nogala-Kalucka M. (2012). Antioxidant activity and phenolic content in three lupin species. J. Food Compos. Anal..

[86] Nimalaratne C., Lopes-Lutz D., Schieber A., Wu J. (2011). Free aromatic amino acids in egg yolk show antioxidant properties. Food Chem..

[87] Untea A.E., Varzaru I., Panaite T.D., Gavris T., Lupu A., Ropota M. (2020). The Effects of dietary inclusion of bilberry and walnut leaves in laying hens’ diets on the antioxidant properties of eggs. Animals.

[88] Gasecka M., Mleczek M., Siwulski M., Niedzielski P., Kozak L. (2015). The effect of selenium on phenolics and flavonoids in selected edible white rot fungi. LWT Food Sci. Technol..

[89] Sae-Lee N., Kerdchoechuen O., Laohakunjit N. (2012). Chemical qualities and phenolic compounds of Assam tea after soil drench application of selenium and aluminium. Plant Soil.

[90] Lei C., Ma Q., Tang Q.Y., Ai X.R., Zhou Z., Yao L., Wang Y., Wang Q., Dong J.Z. (2014). Sodium selenite regulates phenolics accumulation and tuber development of purple potatoes. Sci. Hortic..

[91] Kamboh A.A., Leghari R.A., Khan M.A., Kaka U., Naseer M., Sazili A.Q., Malhi K.K. (2019). Flavonoids supplementation-An ideal approach to improve quality of poultry products. World’s Poult. Sci. J..

[92] Saleh H., Golian A., Kermanshahi H., Mirakzehi M.T. (2018). Antioxidant status and thigh meat quality of broiler chickens fed diet supplemented with α-tocopherolacetate, pomegranate pomace and pomegranate pomace extract. Ital. J. Anim. Sci..

[93] Surai P.F. (2014). Polyphenol compounds in the chicken/animal diet: From the past to the future. J. Anim. Physiol. Anim. Nutr..

[94] Dalia A.M., Loh T.C., Sazili A.Q., Jahromi M.F., Samsudin A.A. (2017). The effect of dietary bacterial organic selenium on growth performance, antioxidant capacity, and Selenoproteins gene expression in broiler chickens. BMC Vet. Res..

[95] Nahariah N., Legowo A.M., Abustam E., Hintono A., Bintoro P., Pramono Y.B. (2014). Endogenous antioxidant activity in the egg whites of various types of local poultry eggs in South Sulawesi, Indonesia. Int. J. Poult. Sci..

[96] Nimalaratne C., Schieber A., Wu J. (2015). Effects of storage and cooking on the antioxidant capacity of laying hen eggs. Food Chem..

[97] Wang J., Jia R., Celi P., Ding X., Bai S., Zeng Q., Mao X., Xu S., Zhang K. (2020). Green tea polyphenol epigallocatechin-3-gallate improves the antioxidant capacity of eggs. Food Funct..

[98] Kullu S.S., Das A., Bajpai S.K., Garg A.K., Yogi R.K., Saini M., Sharma A.K. (2017). Egg production performance, egg yolk antioxidant profile and excreta concentration of corticosterone in golden pheasants (*Chrysolophus pictus*) fed diets containing different levels of green vegetables. J. Anim. Physiol. Anim. Nutr..

[99] McGraw K.J., Hill G.E., Parker R.S. (2005). The physiological costs of being colorful: Nutritional control of carotenoid utilization in the American goldfinch, *Carduelis tristis*. Anim. Behav..

[100] Ahmad H., Tian J., Wang J., Khan M.A., Wang Y., Zhang L., Wang T. (2012). Effects of dietary sodium selenite and selenium yeast on antioxidant enzyme activities and oxidative stability of chicken breast meat. J. Agric. Food Chem..

[101] Tufarelli V., Ceci E., Laudadio V. (2016). 2-Hydroxy-4-methylselenobutanoic acid as new organic selenium dietary supplement to produce selenium-enriched eggs. Biol. Trace Elem. Res..

[102] Lin X., Yang T., Li H., Ji Y., Zhao Y., He J. (2020). Interactions between different selenium compounds and essential trace elements involved in the antioxidant system of laying hens. Biol. Trace Elem. Res..

[103] dos Reis J.H., Gebert R.R., Fortuoso B.F., dos Santos D.S., Souza C.F., Baldissera M.D., de Tavernari F.C., Boiago M.M., Paiano D., Da Silva A.S. (2019). Selenomethionine as a dietary supplement for laying hens: Impacts on lipid peroxidation and antioxidant capacity in fresh and stored eggs. J. Food Biochem..

[104] Wang Z.G., Pan X.J., Zhang W.Q., Peng Z.Q., Zhao R.Q., Zhou G.H. (2010). Methionine and selenium yeast supplementation of the maternal diets affects antioxidant activity of breeding eggs. Poult. Sci..

[105] Gajčević Z., Kralik G., Has-Schön E., Pavić V. (2009). Effects of organic selenium supplemented to layer diet on table egg freshness and selenium content. Ital. J. Anim. Sci..

[106] Kralik Z., Kralik G., Grčević M., Galović D. (2014). Effect of storage period on the quality of table eggs. Acta Agrar. Kaposváriensis.

[107] Radwan Nadia L., Hassan R.A., Qota E.M., Fayek H.M. (2008). Effect of natural antioxidant on oxidative stability of eggs and productive and reproductive performance of laying hens. Int. J. Poult. Sci..

[108] Wang Y., Zhan X., Zhang X., Wu R., Yuan D. (2011). Comparison of different forms of dietary selenium supplementation on growth performance, meat quality, selenium deposition, and antioxidant property in broilers. Biol. Trace Elem. Res..

[109] Vignola G., Lambertini L., Mazzone G., Giammarco M., Tassinari M., Martelli G., Bertin G. (2009). Effects of selenium source and level of supplementation on the performance and meat quality of lambs. Meat Sci..

[110] Zhou X., Wang Y. (2011). Influence of dietary nano elemental selenium on growth performance, tissue selenium distribution, meat quality, and glutathione peroxidase activity in Guangxi Yellow chicken. Poult. Sci..

[111] Franke K.W. (1934). A new toxicant occuring naturlly in certain samples of plant foodstuffs. J. Nutr..

[112] Wang Z.G., Pan X.J., Peng Z.Q., Zhao R.Q., Zhou G.H. (2009). Methionine and selenium yeast supplementation of the maternal diets affects color, water-holding capacity, and oxidative stability of their male offspring meat at the early stage. Poult. Sci..

